# Microplastics in Food: A Review on Analytical Methods and Challenges

**DOI:** 10.3390/ijerph17186710

**Published:** 2020-09-15

**Authors:** Jung-Hwan Kwon, Jin-Woo Kim, Thanh Dat Pham, Abhrajyoti Tarafdar, Soonki Hong, Sa-Ho Chun, Sang-Hwa Lee, Da-Young Kang, Ju-Yang Kim, Su-Bin Kim, Jaehak Jung

**Affiliations:** 1 Division of Environmental Science and Ecological Engineering, Korea University, Seoul 02841, Korea; jinwoo4671@gmail.com (J.-W.K.); thanhdat4196@gmail.com (T.D.P.); abhra@korea.ac.kr (A.T.); 2 FITI Testing & Research Institute, Cheongju 28116, Korea; skhong@fiti.re.kr (S.H.); shchun@fiti.re.kr (S.-H.C.); shlee03@fiti.re.kr (S.-H.L.); dykang@fiti.re.kr (D.-Y.K.); 3 Korea Institute of Analytical Science and Technology, Seoul 04790, Korea; kjy@kiast.co.kr (J.-Y.K.); ksb@kiast.co.kr (S.-B.K.); jh3370@naver.com (J.J.)

**Keywords:** microplastics, seafood, sea salt, density separation, FT-IR, digestion

## Abstract

Human exposure to microplastics contained in food has become a significant concern owing to the increasing accumulation of microplastics in the environment. In this paper, we summarize the presence of microplastics in food and the analytical methods used for isolation and identification of microplastics. Although a large number of studies on seafood such as fish and shellfish exist, estimating the overall human exposure to microplastics via food consumption is difficult owing to the lack of studies on other food items. Analytical methods still need to be optimized for appropriate recovery of microplastics in various food matrices, rendering a quantitative comparison of different studies challenging. In addition, microplastics could be added or removed from ingredients during processing or cooking. Thus, research on processed food is crucial to estimate the contribution of food to overall human microplastic consumption and to mitigate this exposure in the future.

## 1. Introduction

Increased consumption of plastic products in modern society has caused microplastic contamination (i.e., synthetic plastic particles less than 5 mm) in nearly all environmental media [1,2,3,4,5,6,7,8,9,10]. Microplastic accumulation has been reported in beaches, oceans [1,2,3,4], soils and sediments [5,6,7], and freshwater systems [8,9,10]. Therefore, it is likely that global contamination of microplastics will be eventually brought back to our dinner table through consumption of various food items. Although a few studies have quantitatively estimated the microplastic consumption of people from contaminated seafood [11,12,13], salt [14,15], and packaging materials [16,17,18], the extent of people’s microplastic exposure via food consumption remains largely unknown.

Until recently, microplastic analysis has focused on aquatic environments, including organisms for food consumption [11,12,13,19,20,21,22,23,24,25]. However, seafood is not the only source of microplastics. Many other land-based foods might be contaminated with microplastics as well as processed food that is susceptible to microplastic contamination [18]. In many regions, an increasing number of ready-to-eat meals are available for consumers, and microplastics might be added during processing and packaging, despite the original food rarely containing microplastics [26,27]. An example of how packaging materials increase human exposure to microplastics is leaching of micro- and nano-sized plastic particles from a teabag [17]. Additionally, microplastics can be added or removed while processing and cooking raw food for consumption.

The greatest challenge to quantifying microplastic intake via food consumption is the uncertainty of microplastic concentrations in ingredients and cooked food. Microplastic concentrations in food are often very low, requiring tedious pretreatment steps to separate microplastics. Developing standardized experimental protocols for microplastic analysis is also difficult owing to varying food matrices. Although only a few simple steps are needed for isolating microplastics from relatively clean aqueous solutions (e.g., microplastics in sea salts dissolved in water) [14,15], some food matrices contain large quantities of natural polymers and oligomers that are difficult to separate from synthetic plastic particles (e.g., seaweed) [26]. Therefore, microplastic analysis methods for various food items should be compared, and areas requiring further research must be determined.

In this paper, we summarize existing peer-reviewed articles on microplastics in various food ingredients. We also discuss the quantities and types of microplastics as well as analytical methods used for isolating and identifying microplastics from sea salt, fish, shellfish, other ingredients, and processed foods. The advantages and disadvantages of the various analytical methods are compared, and research requirements for improving the assessment of human exposure to microplastics via food consumption are proposed.

## 2. Methods

An increasing number of articles on microplastics have been published recently. Because a keyword search for “microplastic” in 2019 yielded more than 1800 articles in Scopus alone, combinations of keywords were used to increase topic relevance and to narrow down the number of articles to review in two databases, Google Scholar and Scopus, as follows:

“Microplastic” AND {“amphipods” OR “bivalves” OR “clams” OR “crab” OR “mussel” OR “oyster” OR “shrimp” OR “culture” OR “fish” OR “gut” OR “ingestion” OR “wild” OR “beer” OR “canned” OR “honey” OR “milk” OR “salt” OR “seaweed” OR “sugar” OR “teabag”}. Additional articles were added from article citations due to the diversity of food and food processing techniques.

## 3. Results

Most articles on microplastics in food including sea salt and seafood were published during the last decade. Existing research on microplastic occurrence in food including the analytical methods for microplastic separation from various foods, instrumental determination, shapes, and material types are summarized in the following sections.

### 3.1. Microplastic Occurrence in Food

#### 3.1.1. Table Salt

Because table salt is most often produced by the distillation of seawater, it is difficult to avoid microplastics in final sea salt products without further purification steps because seawater contains microplastics [2,28]. Table 1 summarizes the range of microplastics per kilogram of salt along with the analytical methods used. As shown in the table, the concentration of microplastics varied widely from not detected (n.d.) to 5400 particles per kilogram [14,15,29,30,31,32,33,34,35,36]. Hydrogen peroxide (H_2_O_2_) was often used to digest organic matter in the solution after dissolving sea salts [14,15,29,30]. Density separation was usually conducted by using sodium iodide (NaI) up to the solution density of 1.8 g cm^−3^ [30,31]. When microplastics were counted by visual inspection under a dissection microscope with or without staining (e.g., Rose Bengal) [32], the resulting concentrations were usually greater than those counted under a microscope coupled with Fourier-transform infrared (FT-IR) spectroscopy (Table 1), implying potential false-positive counting. When large pore size (149 μm) was used [31], significantly lower microplastic concentrations were observed both in sea salts and lake salts. However, no significant differences were identified in microplastic concentrations owing to the experimental size cutoff at a lower range (0.2–11 μm). This might be because the detection of microplastics less than 10 μm would be very difficult using stereomicroscope coupled with FT-IR spectroscopy [37]. The levels of microplastics in rock salts and lake salts were not significantly different from those in sea salts, although they contained more fibers [14,15,30,31,32,33,36]. Thus, further investigation is required to minimize microplastic contamination during the production of table salt from sources other than seawater.

#### 3.1.2. Fish and Shellfish

In the last decade, researchers have identified the presence of microplastics in fish and shellfish captured in the wild and obtained from aquaculture farms or markets, as summarized in Table 2 and Table 3 [23,25,38,39,40,41,42,43,44,45,46,47,48,49,50,51,52,53,54,55,56,57,58,59,60,61,62,63,64,65,66,67,68,69,70,71,72,73,74,75,76,77,78,79,80,81,82,83,84,85,86,87,88,89,90,91,92,93,94,95,96,97,98,99]. However, it is unclear whether aquaculture activity increases the possibility of microplastic contamination in fish. In cases where fish or shellfish were obtained near the coastline, their levels of microplastics were good indicators of microplastic contamination in the coastal environment.

Biological matrices were digested and removed from fish samples using various pretreatment methods. For large fish, the stomach and/or gastrointestinal tract (GIT) was isolated and digested to detect microplastics [9,25,38,39,40,41,42,43,44,45,46,47,48,49,50,51,52,53,54,55,56,57,58,60,61,62,63,65,66,67,68,69,70,72,73,74,75]. Unlike the digestion method used for sea salts, various chemicals were used to decompose organic materials, including potassium hydroxide (KOH) [9,22,23,38,45,47,48,53,55,56,58,62,64,71,74,75], sodium hydroxide (NaOH) [42,68,90], hydrogen peroxide (H_2_O_2_) [22,39,40,41,52,57,59,61,69,73,74], Fenton’s reagent (Fe^2+^ ion with H_2_O_2_) [44], nitric acid (HNO_3_) [22,43,59,65], perchloric acid (HClO_4_) [22,43], and digestive enzymes [46]. The time required for digestion varied depending on the amount and quality of the samples. Higher digestion temperatures could shorten the required time, but they increase the possibility for thermoplastic loss [100]. Microscopic analysis coupled with FT-IR spectroscopy was the most popular method for chemical identification of microplastics [9,25,39,40,41,42,44,45,46,47,48,50,51,52,53,54,55,56,57,58,59,61,64,69,70,71,73,74,75]. Raman spectroscopy was also applied [59,62] due to its enhanced performance with smaller particles [100]. Visual inspection under a microscope with or without staining was less popular, most likely because of the complexity of the sample matrices and the potential for false-positive identification [43,49,60,63,65,66,67,68,72]. Further, scanning electron microscopy (SEM) was used to identify smaller microplastic particles [22,38].

Microplastics were rarely found in edible tissues, such as muscle [9,22,59,64,71], but they were predominantly found in digestive tracts [9,22,23,25,38,39,40,41,42,43,44,45,46,47,48,49,50,51,52,53,54,55,56,57,58,60,61,62,63,65,66,67,68,69,70,72,73,74,75]. Because it is unclear how microplastics are transported to edible tissues, human exposure to microplastics due to the consumption of edible fish tissue requires further evaluation. Although the results are difficult to compare from the studies listed in Table 2 owing to a variety of experimental protocols for isolating and identifying microplastics, the reported range of microplastics in stomach contents or GIT were n.d.—35 items per individual fish or n.d.—19.2 items per gram. Sources of fish (i.e., aquaculture, wild, or from market) did not exhibit any significant deviations in microplastic concentrations, although a direct comparison of studies might not be appropriate.

Gill tissue has been found to be another important entry point for microplastics in fish [9,69,72]. Although microplastics from the water can accumulate in gill tissue during ventilation, it is unlikely that microplastics are introduced into the circulatory system since they were not identified in soft and edible tissues of the same fish [9]. Further investigation of the adverse effects of microplastics in fish gills versus those by ingestion is required because the effects of microplastics depend on the point of entry.

Microplastics were also extensively monitored in shellfish, such as blue mussels, shrimps, and clams [11,20,40,45,52,59,76,77,78,79,80,81,82,83,84,85,86,87,88,89,90,91,92,93,94,95,96,97,98,99] (Table 3). Unlike fish, soft tissues were dissected and digested to separate microplastics in mussels, clams, and oysters. The predominant oxidation and digestion methods used for shellfish were oxidation using H_2_O_2_ or digestion in acidic (HNO_3_) or basic (KOH) solutions. Shellfish have traditionally been used to monitor environmental contaminants in coastal areas [101,102] and were thought to be suitable model indicator organisms for microplastics as emerging contaminants. The majority of microplastic concentrations in blue mussels, which are the most extensively studied species, were less than 1 item per gram [11,20,21,48,78,81,83,84]. However, over a hundred microplastic particles were found in the organic tissues of mud crabs, although the particles were not chemically identified using spectroscopic methods [85], requiring further observation of the levels of microplastics in various shellfish.

Before shellfish is cooked, it is recommended that the contents of their digestive systems be depurated. Further, pretreatment before consumption is important to estimating human exposure to microplastics from shellfish because depuration of mussels reduces microplastics in their body [103].

#### 3.1.3. Processed Foods

Microplastics were also isolated from various processed foods (Table 4). They were investigated in liquids such as beer [32,104], honey [105,106,107], and milk [108]. The concentrations ranged from n.d. to several hundred particles per liter [32,104,105,106,107,108]. The high concentrations of microplastics in beer samples require further confirmation because staining and visual counting may have overestimated the number of particles [32,104]. Although the honey samples were oxidized using 30% H_2_O_2_, a large number of suspected particles, up to thousands per kilogram, were observed [105,106,107]. As all the individual particles were not chemically analyzed with FT-IR, the occurrence of microplastics in honey requires further evaluation using more advanced methods. Milk might be contaminated with microplastics during processing; therefore, determining how microplastics are introduced into the final milk products is important.

Although sugar contains nearly as much microplastic as sea salt [105], the only study on microplastics from sugars did not use spectroscopic identification methods, and it might include other particles rather than microplastics. Sugar might also be contaminated with microplastic during processing, requiring further investigations.

Dried food such as land animal-based Chinese traditional medicine [109], processed seafood such as sardines and sprats [27], seaweed [26], dried fish [24], and tea in teabags [17] are also contaminated with microplastics. The high microplastic concentrations in Chinese traditional medicine is due to high microplastic levels in the source animals. In many places, people consume food or medicine that are easily contaminated by microplastics, and studies should be conducted to reflect local consumption patterns. Dried seafood is usually consumed whole. Thus, microplastics in dried seafood are more important than those reported in the GIT of fish (Table 2) from the human exposure perspective. However, it is unclear how the contamination of dried seafood occurred and could be mitigated. The contribution from the organisms in addition to processing techniques, such as drying and packaging, should be evaluated to minimize the microplastic concentration.

One study evaluated high concentrations of anthropogenic particles in hot water from teabags [17]. Over a thousand micrometer-sized particles and millions of sub-micrometer-sized particles were identified under SEM and X-ray photoelectron spectroscopy (XPS), respectively, from only 1 mm^2^ of the teabag surface. However, not all particles were identified as microplastics [17]. Thus, the microplastic concentration in teabags should be determined and exposure be reduced as smaller particle sizes are more likely to affect organs after ingestion.

### 3.2. Analytical Methods

#### 3.2.1. Pretreatment Methods

As summarized in Table 1, Table 2, Table 3 and Table 4, various pretreatment methods were employed to isolate microplastics. Although washing with deionized water and then visual inspection with or without staining is convenient for clean matrices [32,34], false-positive detection of microplastics is challenging to avoid. As shown in Table 1 and Table 4, the concentration of microplastics obtained after staining was higher than those obtained by using other methods. In liquid samples, such as dissolved sea salt and honey, H_2_O_2_ was effective for the removal of other organic materials that inhibit microplastic detection. The typical mass concentration of H_2_O_2_ was 30% (v/v). Digestion temperatures and times ranged from 50–70 °C and 12–96 h, respectively [100]. A longer digestion at higher temperature is beneficial for eliminating impurities that impede microplastic detection. However, certain polymeric materials such as polyacrylate (PA) and polyvinyl chloride (PVC) can decompose and nylon 66 may melt and be lost during digestion at high temperatures [100,110,111]. Another popular oxidation method is the use of Fenton’s reagent. This method is suggested by the National Oceanic and Atmospheric Administration, USA, for marine organisms [112], although the method needs to be tested for a diversity of organic matrices.

Digestion with alkaline solutions such as KOH and NaOH have predominantly been used for digesting fish and shellfish (Table 2 and Table 3). It is advantageous for destroying proteins and other soft tissues. Suitable extraction recovery was found for polyethylene terephthalate (PET) and high-density polyethylene [113]. However, pH-sensitive polymers such as nylon and polyester can be disrupted at high pH [114]. Various strong acid solutions (e.g., HNO_3_, HCl/HNO_3_, and HClO_4_) have been used to digest the soft tissues of fish, mussels, and other organisms [19,20,31,94,115,116]. Similar to strong basic solutions, the tissues were successfully decomposed, although low pH also led to the decomposition of pH-sensitive polymers.

Several digestive enzymes such as proteinase, trypsin, and collagenase have also been tested [19,110,117,118,119]. Because these enzymes are effective at moderate pH and redox conditions and specifically degrade proteins and other biological polymers that can be digested by organisms, damage from microplastics can be minimized. However, these enzymes are much more expensive than inorganic oxidants and acids and/or bases and do not work well on high-density organic material. Thus, further validation of enzymatic methods is required. In some studies, enzymatic digestion was augmented with other reactants to enhance efficiency [19,120,121]. For example, Löder et al. [121] proposed a basic enzymatic purification protocol in which protease, cellulase, and chitinase were sequentially used with H_2_O_2_. Microplastics were successfully isolated through multiple steps of filtration, digestion, and rinsing, but the method is time consuming and poses the risk of microplastic loss during repeated processing. Similarly, Mintenig et al. [120] used an enzyme-oxidative procedure wherein the solution was sequentially washed with sodium dodecyl sulfate, protease, lipase, cellulase, H_2_O_2_, and chitinase solutions. Although the method was used to remove organic matter in wastewater, these repeated steps can be applied to complex food matrices.

#### 3.2.2. Microplastic Identification

The two predominant methods used for microplastic identification in food were visual inspection under dissection microscope with or without staining and the absorption or reflection of IR with FT-IR or Raman spectroscopy (Table 2, Table 3 and Table 4). Although it is a destructive method, thermal decomposition coupled with gas chromatography–mass spectrometry (GC-MS) attracted attention for quantitative analysis of microplastic mass in environmental samples [122,123,124,125]. As chemical fingerprints are used after pyrolysis, this method can also be used for simultaneous determination of plastic materials as well as major additives.

### 3.3. Material Type, Shape, and Size

#### 3.3.1. Plastic Materials in Food

**Figure 1:**
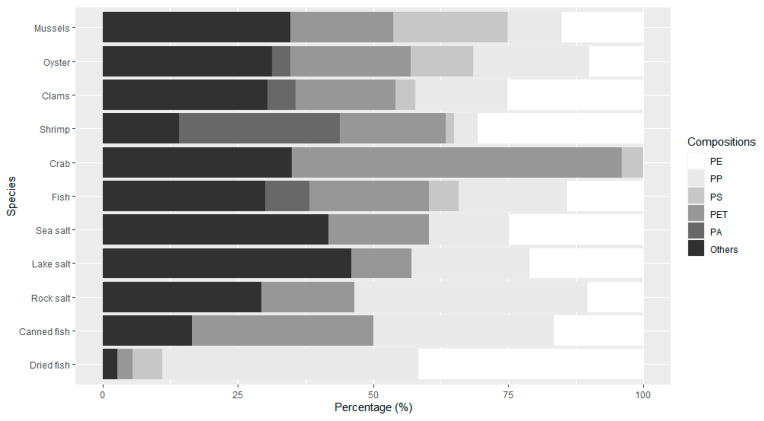
Fractions of plastic materials identified in seafood and salt. Data from References [11,14,15,21,24,29,30,34,39,40,45,47,48,50,51,52,62,71,74,75,76,77,78,80,81,86,90,91,95,96,98,99]. (PE: polyethylene, PP: polypropylene, PS: polystyrene, PET: polyethylene terephthalate, PA: polyacrylate).

Thermoplastics (i.e., polyethylene (PE), polypropylene (PP), polystyrene (PS), and PET) comprised the majority of microplastics found in food [11,14,15,21,24,30,39,45,47,48,50,51,52,62,71,74,76,77,78,80,81,86,95,96,98,99]. Figure 1 summarizes the average fractions of plastic materials in representative food items. In all foods, PE, PP, PS, and PET (including polyesters) account for more than 50% of microplastics. Cellophane was found to be dominant in table salt [35], fish [40], and clams [90,91]. However, cellophane is a thin regenerated form of cellulose and is difficult to discern from naturally occurring plant-derived polymers through spectroscopic identification of smaller-sized particles. Polyethersulfone (PES) was found to be dominant, accounting for 80% of oysters in China [75] and 30% in Indian sea salts [29], but it was rarely found in other studies, implying the need for further investigation.

#### 3.3.2. Microplastic Shape and Size

**Figure 2:**
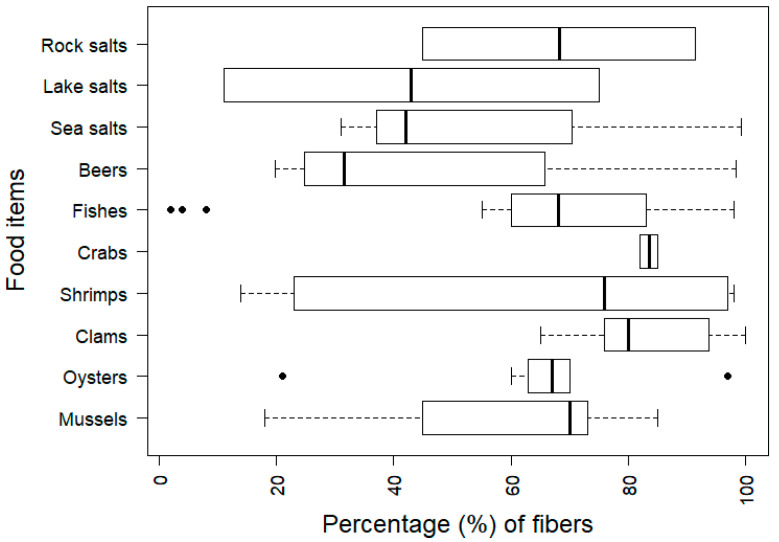
Boxplot of microplastic fibers including filaments in different food items representing 5, 25, 50, 75, and 95 percentile values. Filled circles indicate outliers. Data from References [14,23,24,25,29,30,31,32,38,39,40,45,47,48,50,52,62,68,71,74,75,76,77,78,79,80,81,85,86,87,88,89,90,91,94,95,96,97,98,99].

Microplastic particles are often classified as fibers, fragments, pellets, or films [3,50,52,68,87,98,126]. Fibers are critical because they are thought to cause toxic effects at lower doses than spherical particles [127,128,129]. Fibers including particles classified as “filaments” were dominant in many food items [23,25,30,31,32,39,40,45,48,50,62,68,71,74,75,77,78,79,80,85,86,87,89,90,91,94,96,97,99]. Figure 2 shows a boxplot describing the percentage of fibers in various food items. For fish, only microplastics isolated from edible tissue were counted. The percentage of fibers in isolated microplastics was more than 50% in various food items. For example, the fraction of fibers reached almost 100% of microplastics in sea salts [32] and edible tissues of fish [71,74] and shellfish [74,91,97]. However, a low fraction (<20%) of fibers was identified in lake salts [14], edible tissues of fish [38,47], mussels [81], shrimp [98], and dried fish [24]. This variation in the percentage of fibers can be attributed to the sources of microplastics, differences in food matrices, and diverse analytical methods used. As fibers can be lost more easily than spherical or elliptical particles during digestion and filtration [130], extra care is required to recover fibers from food matrices.

## 4. Discussion

### 4.1. Analytical Challenges

Although tremendous efforts have been made in the last decade to identify microplastics in food, standardized experimental protocols have not been attained. Among many experimental protocols attempted, the most common and reliable methods are oxidative digestion and filtering and spectroscopic confirmation with FT-IR when the particle size is greater than 50 μm [131]. Recent advances in mapping suspect particles on a filter and automatically scanning them remarkably reduce the required labor and experimental time [132,133]. However, the overall time required for microplastic isolation and identification still does not satisfy the analytical requirements.

To analyze human exposure to microplastics, the level of microplastic exposure to smaller particles should be determined because it has been reported that toxic effects of microplastics on aquatic species depend on particle size [134,135]. However, the predominant methods for identifying microplastics using FT-IR or Raman spectroscopy are only able to confirm microplastics with the size greater than 10 μm [131], whereas toxic effects were mostly observed for much smaller particles, making a gap between exposure and effect assessment. Levels of smaller microplastics in food might be indirectly estimated if the typical microplastic particle size distribution is identified. Extrapolating the level of smaller microplastics from chemically identified larger microplastics would fill this gap. However, it is still unclear whether microplastic particle size distribution follows the Power law. While a few studies reported that smaller microplastics are more abundant than larger microplastics following the Power law [136,137], other studies observed that the most abundant particle size is greater than the experimental thresholds [3,138]. Further, studies on microplastic particle size distribution in various types of food are scarce, requiring investigation on size distribution.

In addition to counting and confirming microplastics, thermal analysis has attracted attention recently, although it is a destructive method [123,124,125]. Particulate matter concentrations in air and water are traditionally reported as mass per volume and used as a dose metric in health risk assessments. Therefore, quantifying microplastics as mass per volume or mass per food item could be an alternative and practical approach to represent the concentration of microplastics. Because food matrices contain many polymeric materials that break into small molecules that might interfere with indicator species of microplastics, gas chromatography–mass spectrometry is coupled with pyrolysis. Thus, appropriate pretreatment techniques are crucial for the application of pyrolysis for mass determination of microplastics in food. Table 5 summarizes the advantages and disadvantages of existing microplastic identification methods used in the literature.

Until recently, the predominant biological matrices of fish and shellfish were extensively investigated. However, it is unclear whether established methods are directly applicable to other important food items, especially those containing large amounts of natural polymeric materials. For example, chilis and bean pastes are widely used in Korean food, and these products can be contaminated by their ingredients or during processing. Food originating from plants contain high fractions of cellulose. Although the basic enzymatic purification protocol including cellulase and other digestive enzymes has been proposed for isolating microplastics in plankton samples [121], enzyme treatments are usually more expensive than chemical treatments and often not conventional, as summarized in Table 6. Thus, further investigation is required for complex food matrices.

### 4.2. Estimation of Human Microplastic Exposure via Food Consumption

Human exposure to microplastics through food consumption can be estimated using a simple exposure equation:

TDI = Σ*_i_* (IR*_i_* EF*_i_* C*_i_*)/BW (1)

where TDI is the total daily intake (items or mass per kg-d), IR*_i_* is the intake rate of food item *i* (g of food item *i* per serving), EF*_i_* is the exposure frequency (servings per day), C*_i_* is the number or mass concentration of microplastics in food item *i* (items or mass per g), and BW is body weight (kg). Exposure parameters, IR*_i_* and EF*_i_*, are often available from national nutrition databases. However, these data are often based only on the final food item, not the ingredients, whereas microplastic concentration is usually evaluated for each ingredient. Preparation and cooking before consumption can significantly increase or decrease the actual microplastic concentrations in consumed foods [103,117]. Thus, a comparison of different analyses from food ingredients to final food items is necessary to mitigate human exposure to microplastics through food consumption.

The microplastic concentration in food (C*_i_*) is also a complex metric. As reviewed in this paper, the occurrence of microplastics is usually presented as the number of particles per mass of the food item. However, it is well-acknowledged that various shapes and sizes of microplastics are also important for determining the adversity of microplastics in humans. Smaller and fiber-type particles are often regarded as more dangerous than larger and fragment-type particles [134,135] and nano-sized microplastics may cross barriers in digestive systems [139]. As shown in Figure 2, the fraction of fiber-type microplastics varies among different food items and studies, even for the same food items, and there are gaps between the level of microplastics in food items and that causing adverse outcomes in animal studies; thus, the adverse health effects from consumption of microplastics are difficult to assess. As the maximum allowable intake rate of fibers is suspected to be much lower than that of spheres and fragments, although further investigation is needed [134,135], studies that monitor concentrations of microplastics in various food items should be conducted carefully to evaluate the presence of fiber-type microplastics in food.

It should be also noted that human exposure to microplastics could be dominated by other routes such as inhalation of microfibers [140,141]. Although most indoor particles are biological origin, on average, 4% of identified particles were synthetic fragments and fibers [141]. Because the adversity of microplastics depend on the routes of exposure, the contribution of food consumption to the overall exposure to microplastics needs to be considered within a comprehensive exposure assessment framework.

Given that counting microplastics is a time-consuming task, there is a trend to report C*_i_* based on microplastic mass per mass using thermal analysis [122,123,124,125]. As noted in Table 5, the disadvantages of the thermal method are that (1) it is destructive and (2) specific information on particle size and morphology is unavailable. If thermal analysis will be used to evaluate C*_i_* in food, the typical size distribution and appropriate dose–response relationship should be identified based on microplastic mass.

## 5. Conclusions

Despite the diversity of food consumed in different geographic regions of the world, only limited studies have been conducted on the presence of microplastics in food. The majority of studies analyzed the concentration, materials, morphology, and size of microplastics in salt, fish, and shellfish. Owing to the lack of studies on other food items, the overall human microplastic exposure via food consumption is difficult to estimate and compare with other routes of exposure such as inhalation of micro-sized particles. Although the last decade has shown significant advances regarding this issue, experimental methods for isolating and identifying microplastics in food still need improvement for the appropriate recovery of microplastics in various food matrices and the quantitative comparison of studies. Two current approaches—counting microplastics with microscopy and destructive microplastic detection with thermal analysis—can be complementary. In addition, contamination and decontamination of microplastics during food processing and cooking are important as microplastic exposure of people is primarily from the consumed final products, not on their ingredients.

## Figures and Tables

**Table 1:** Analytical methods and microplastic concentrations in salts.

Salt Sample	Analytical Methods			Concentration (Particles kg^−1^)	References
	Digestion/Density Separation	Filtration Pore Size (μm)	Identification		
Sea salt from 16 countries	17.25% H_2_O_2_	2.7	Microscope/FT-IR	n.d. *–1674	[14]
Sea salt from India	30% H_2_O_2_	0.45	Microscope/FT-IR	56(±49)–103(±39)	[29]
Sea salt from China	30% H_2_O_2_	5	Microscope/FT-IR	550–681	[15]
Sea salt from Turkey	30% H_2_O_2_/1.8 g cm^−^^3^ NaI	0.2	Microscope/Raman	16–84	[30]
Sea salt from 6 countries	1.5 g cm^−^^3^ NaI	149	Microscope/Raman	n.d.–10	[31]
Sea salt from 8 seas/oceans	Rose Bengal	11	Dissection microscope	46.7–806	[32]
Sea salt from Spain	distilled water/centrifuge	5	Microscope/FT-IR	50–280	[33]
Sea salt from Italy and Croatia	Deionized water	0.45	Microscope/FT-IR	n.d.–19800	[34]
Sea salt from Italy and Croatia	Deionized water	0.2	Microscope/FT-IR	70–320	[35]
Sea salt from Taiwan	Filtered water	5	Microscope/FT-IR	2.5–20	[36]
Lake salt from China	30% H_2_O_2_	5	Microscope/FT-IR	43–364	[15]
Lake salt from China and Senegal	17.25% H_2_O_2_	2.7	Microscope/FT-IR	28–462	[14]
Lake salt from Turkey	30% H_2_O_2_/1.8 g cm^−^^3^ NaI	0.2	Microscope/Raman	8–102	[30]
Lake salt from Iran	1.5 g cm^−^^3^ NaI	149	Microscope/Raman	1	[31]
Rock salt from 8 countries	17.25% H_2_O_2_	2.7	Microscope/FT-IR	n.d.–148	[14]
Rock salt from Turkey	30% H_2_O_2_/1.8 g cm^−^^3^ NaI	0.2	Microscope/Raman	9–16	[30]
Rock salt from 2 countries	Rose Bengal	11	Dissection microscope	113–367	[32]
Rock salt from Taiwan	Filtered water	5	Microscope/FT-IR	12.5	[36]
Rock/well salt from China	30% H_2_O_2_	5	Microscope/FT-IR	7–204	[15]
Well salt from Spain	Distilled water/centrifuge	5	Microscope/FT-IR	115–185	[33]

* n.d.: not detected.

**Table 2:** Analytical methods and microplastic concentrations in fish.

Species	Analytical Methods			Concentration	References
	Digestion/Density Separation	Filtration Pore Size (μm)	Identification		
13 species (US) (M) ^1^	GIT ^2^; 10% KOH (v/v)	-	Microscope/SEM	n.d.–10/fish	[38]
11 species (Indonesia) (M)	GIT; 10% KOH (v/v)	-	Microscope/SEM	n.d.–21/fish	[38]
Flathead grey mullet (*M. cephalus*) (M)	GIT; 30% H_2_O_2_ (v/v); NaCl 1.2 g mL^−^^1^	11	Microscope/FT-IR	4.3/fish	[39]
11 species (M)	GIT; 30% H_2_O_2_ (v/v); NaCl 1.2 g mL^−^^1^	5	Microscope/FT-IR	0.2–17.2/g	[40]
13 species (M)	GIT; 15% H_2_O_2_ (v/v); NaCl 1.2 g mL^−^^1^	0.45	Microscope/FT-IR	1.32 ± 0.48/fish	[41]
Nile perch and Nile tilapia (M)	GIT digested with NaOH	250	Microscope/FT-IR	-	[42]
9 species (M)	GIT; HNO_3_:HClO_4_ (1:5)	-	Stereomicroscope	-	[43]
9 species (M)	GIT; FeSO_4_ 0.05M/30% H_2_O_2_; NaCl	8	Microscope/FT-IR	5.0 ± 2.5/fish	[44]
26 species (M)	Stomach content, washed with distilled water	-	Microscope/FT-IR	0.27 ± 0.63/fish	[25]
4 species (M)	Whole sample; 10% KOH	2	Microscope/SEM	1.00 ± 0.96/g	[23]
*A. latus*; *K. punctatus* (M)	GIT; 10% KOH; NaCl 1.2 g mL^−1^	-	Microscope/FT-IR	0.49–1.26/g	[45]
4 species (W)	GIT, proteinase-K	0.7	Microscope/FT-IR	-	[46]
Japanese anchovy (*E. japonicus*) (W)	GIT; 10% KOH	-	Microscope/FT-IR	2.3 ± 2.5/fish	[47]
European anchovies (*E. encrasicolus*) (W)	GIT; 10% KOH (v/v)	GF/A	Microscope/FT-IR	2.5 ± 0.3/fish	[48]
Catfish (*H. littorale*) (W)	GIT content, washed with distilled water	63	Dissection microscope	1–24/fish	[49]
10 species (W)	GIT, cut open and observed	-	Microscope/FT-IR	1–15/fish	[50]
Demersal fish (3 species) (W)	Gut content, suspended in distilled water	500	Microscope/FT-IR	0.03 ± 0.18/fish	[51]
Pelagic fish (2 species) (W)	Gut content, suspended in distilled water	500	Microscope/FT-IR	0.19 ± 0.61/g	[51]
4 species (W)	GIT; 15% H_2_O_2_(v/v)	-	Microscope/FT-IR	3.2 ± 1.9/fish	[52]
Black rabbitfish (*S. fuscescens*) (W)	GIT; 10% KOH (v/v)	8	Microscope/FT-IR	0.6/g	[53]
Easter Island flying fish (*C. rapanouiensis*) W)	GIT content, washed with distilled water	100	Microscope/FT-IR	1.5 ± 0.7/fish	[54]
Yellowfin tuna (*T. albacares*) (W)	GIT content, washed with distilled water	100	Microscope/FT-IR	n.d.–5/g	[54]
Cod (W)	GIT; 10% KOH (v/v)/citric acid	2.7	Microscope/FT-IR	0.23/fish	[55]
Saithe (W)	GIT; 10% KOH (v/v)/citric acid	2.7	Microscope/FT-IR	0.28/fish	[55]
5 species (W)	GIT; 10% KOH (v/v)	200	Microscope/FT-IR	1–4/fish	[56]
28 species (W)	GIT; 35% H_2_O_2_	26	Microscope/FT-IR	1–35/fish	[57]
4 species (W)	GIT; 10% KOH (v/v)	20	Microscope/FT-IR	0.005/fish	[58]
Brown trout (*S. trutta*) (W)	GIT, proteinase K	1.2	Stereomicroscope/Raman/hot needle test/FT-IR	1.96/fish	[59]
Atlantic cod (*G. morhua*) (W)	GIT, washed with distilled water	1000	Dissecting microscope	n.d.–2/fish	[60]
34 species	GIT; 15% H_2_O_2_ (v/v)	63	Microscope/FT-IR	2.4 ± 0.2/fish	[61]
Small-spotted catshark (*S. canicular*) (W)	GIT; 10% KOH (v/v); NaCl	8	Microscope/Raman	0.7/fish	[62]
Herring (*C. harengus*) (W)	GIT, washed with deionized particle-free water	-	Visual inspection	1/g	[63]
Variety (W)	KOH/NaCl	20	FT-IR	-	[64]
6 species (W)	GIT; 10% KOH (v/v)	45	Microscope/FT-IR	22.0 ± 14.6/fish	[9]
6 species (W)	Gill	45	Microscope/FT-IR	8.3 ± 6.0/fish	[9]
6 species (W)	Flesh; 10% KOH (v/v)	45	Microscope/FT-IR	n.d./fish	[9]
2 species (W)	GIT; 65% HNO_3_ (v/v); NaCl solution	-	Microscope	9.6 ± 3.3 (Muara Kamal)8.8 ± 2.7 (Marunda)	[65]
*Acanthopagrus latus*; *Konosirus punctatus* (W)	GIT; 10% KOH (v/v); NaCl 1.2 g mL^−^^1^	1.6	Microscope/FT-IR	1.26 ± 0.34/g	[45]
6 species (W)	Stomach; removed	-	Microscope	1–83/fish	[66]
3 species (W)	Stomach	-	Visual inspection	3.4 ± 2.4/fish	[67]
Red mullets *(M. barbatus*) (W)	Stomach content; 1 M NaOH	-	Microscope	1.75 ± 1.14/fish	[68]
Dogfish *(S. canicula*) (W)	Stomach content; 1 M NaOH	-	Microscope	1.20 ± 0.45/fish	[68]
26 species (W)	Stomach contents, washed with distilled water	-	Microscope/FT-IR	0.27 ± 0.63/fish	[25]
5 species (W)	Tissue; 35% H_2_O_2_/4% KOH/HNO_3_:HClO_4_ (4:1 v:v); NaI 1.7g mL^−^^1^	2	Microscope/SEM	0.16–1.5/g	[22]
12 species (W)	Gut; 30% H_2_O_2_ (v/v)	20	Microscope/FT-IR	0.1–8.8/g	[69]
2 species (W)	Gill	20	Microscope/FT-IR	0.1–5.2/g	[69]
32 species (W)	GIT; 10% KOH (v/v); NaCl 1.2g mL^−^^1^	20	Microscope/FT-IR	2.83 ± 1.84/fish	[70]
Kammal thryssa *(T. kammalensis*) (W)	Tissue; 10% KOH (v/v)	8	Microscope/FT-IR	11.19 ± 1.28/g	[71]
Gizzard shad *(D. cepedianum*) (W)	GIT; KOH; NaCl	0.8	Microscope	3/fish	[72]
Gizzard shad *(D. cepedianum*) (W)	Gill	0.8	Microscope	4/fish	[72]
Largemouth bass *(M. salmoides*) (W)	GIT; KOH; NaCl	0.8	Microscope	16/fish	[72]
Largemouth bass *(M. salmoides*) (W)	Gill	0.8	Microscope	9/fish	[72]
Milkfish *(C. chanos*) (A)	GIT; 65% HNO_3_ (v/v); NaCl		Microscope	9.1 ± 3.0/g	[65]
Milkfish *(C. chanos*) (A)	GIT; 30% H_2_O_2_ (v/v)	-	Microscope/FT-IR	2.3 ± 2.3/fish	[73]
Milkfish *(C. chanos*) (A)	GIT; 30% H_2_O_2_ (v/v)	-	Microscope/FT-IR	1.3 ± 1.0/fish	[73]
Yellow croaker *(L. crocea*) (A)	GIT; 10% KOH (v/v)/30% H_2_O_2_	0.7	Microscope/FT-IR	0.008 ± 0.006/g	[74]
Spotted sardine *(K. punctatus*) (A)	GIT; 10% KOH (v/v)/30% H_2_O_2_	0.7	Microscope/FT-IR	0.044 ± 0.025/g	[74]
12 species (A)	GIT; 10% KOH (v/v)	5	Microscope/FT-IR	3.6 ± 0.4/g	[75]

n.d.: not detected, ^1^ M: bought from market; W: caught in wild; A: obtained from aquaculture farm; ^2^ GIT: gastrointestinal tract.

**Table 3:** Analytical methods and microplastic concentrations in shellfish.

Species	Analytical Methods			Concentration (Particles g^−^^1^)	References
	Digestion/Density Separation	Filtration Pore Size (μm)	Identification		
Blue mussel (*M. edulis*) (M) ^1^	Soft tissue; 30% H_2_O_2_ (v/v)	5	Microscope/FT-IR	3.69–9.16	[76]
Blue mussel (*M. edulis*) (M)	Soft tissue; 30% H_2_O_2_ (v/v); NaCl 1.2 g mL^−^^1^	5	Microscope/FT-IR	0.9–1.4	[77]
Blue mussel (*M. edulis*) (M)	Soft tissue; 10% KOH (v/v)	20	Microscope/FT-IR	n.d.–0.35	[78]
Blue mussel (*M. edulis*) (M)	Soft tissue; HNO_3_:HClO_4_ (4:1 v:v)	Qualitative filter	Stereo microscope	0.35	[20]
Blue mussel (*M. edulis*) (M)	Soft tissue, Corolase^®^ 7089 enzyme mixture	0.8	Microscope/FT-IR	0.74 ± 0.125	[11]
11 species (M)	Soft tissue; H_2_O_2_ 30% (v/v); NaCl 1.2 g mL^−^^1^	5	Microscope/FT-IR	2.1–10.5	[79]
3 species (M)	Soft tissue; 10% KOH (v/v); NaCl 1.2 g mL^−^^1^	1.6	Microscope/FT-IR	0.30 ± 0.10	[45]
Blue mussel (*M. edulis*) (M)	Soft tissue; 30% H_2_O_2_ (v/v); NaCl 1.2 g mL^−^^1^	5	Microscope/FT-IR/SEM	2.7	[80]
Blue mussel (*M. edulis*) (M)	Soft tissue; 30% H_2_O_2_ (v/v); NaCl 1.2 g mL^−^^1^	5	Microscope/FT-IR	0.7–2.9	[77]
Blue mussel (*M. edulis*) (M)	Soft tissue; 10% KOH (v/v); KI (50%, m/v)	12	Microscope/FT-IR	0.23 ± 0.20	[81]
Blue mussel (*M. edulis*) (M)	Soft tissue; 65% HNO_3_/30% H_2_O_2_ (v/v)	1.2	Microscope/Raman/hot needle test/FT-IR	4–10	[59]
Blue mussel (*M. edulis*) (M)	Soft tissue; 65% HNO_3_/30% H_2_O_2_ (v/v)	1.2	Microscope/Raman/hot needle test/FT-IR	1–4	[59]
Blue mussel (*M. edulis*) (M)	Soft tissue; HNO_3_:HClO_4_ (4:1 v:v)	Qualitative filter	Stereo microscope	0.26–0.51	[20]
Blue mussel (*M. edulis*) (M)	Soft tissue, Corolase^®^ 7089 (AB Enzyme GmbH, Darmstadt, Germany) enzyme mixture	0.8	Microscope/FT-IR	0.086 ± 0.031	[11]
Mediterranean mussel (*M. galloprovincialis*) (W)	Soft tissue; 15% H_2_O_2_ (v/v)	-	Microscope/FT-IR	1–2/individual	[52]
Variety (W)	Soft tissue; 30% H_2_O_2_ (v/v); NaCl 1.2 g mL^−^^1^	0.8	Dissection microscope	35/individual	[82]
Blue mussel (*M. edulis*) (M)	Soft tissue; 30% H_2_O_2_ (v/v); NaCl 1.2 g mL^−^^1^	5	Microscope/FT-IR/SEM/stain	1.6	[80]
Blue mussel (*M. edulis*) (M)	Soft tissue; 10% KOH (v/v)	12	Microscope/Raman	0.15 ± 0.06	[21]
Blue mussel (*M. edulis*) (M)	Soft tissue; 69% HNO_3_ (v/v)	5	Microscope/Raman	0.36 ± 0.07	[83]
Variety (A)	Soft tissue; 30% H_2_O_2_ (v/v); NaCl 1.2 g mL^−^^1^	0.8	Dissection microscope	75/individual	[82]
Pacific oyster (*C. gigas*) (M)	Soft tissue; 30% H_2_O_2_ (v/v); saline solution 25%	5	Raman/FT-IR	0.077	[84]
Pacific oyster (*C. gigas*) (M)	Soft tissue; 10% KOH (v/v)	-	Microscope	n.d.–2	[38]
Pacific oyster (*C. gigas*) (M)	Soft tissue; 10% KOH (v/v)	20	Microscope/FT-IR	n.d.–0.19	[78]
Pacific oyster (*C. gigas*) (M)	Soft tissue; 69% HNO_3_ (v/v)	5	Microscope/Raman	0.47 ± 0.16	[83]
Eastern oyster (*C. virginica*) (W)	Soft tissue; 30% H_2_O_2_ (v/v)	0.45	Microscope	3.84 ± 3.39	[85]
Pacific oyster (*C. gigas*) (W)	Soft tissue; 10% KOH (v/v); KI solution (50%, m/v)	12	FT-IR	0.18 ± 0.16	[81]
Sydney rock oyster (*S. glomerate*) (W)	Soft tissue; 10% KOH (v/v); NaI	1	Microscope/FT-IR/stain	0.15–0.83	[86]
Spiny oyster (*S. spinosus*) (W)	Soft tissue; 10% KOH (v/v)	1.6	Microscope/Raman	0.45 ± 0.3	[48]
Atlantic pearl-oyster (*P. radiata*) (W)	Soft tissue; 30% H_2_O_2_ (v/v)	25	Microscope/FE-SEM/FT-IR/hot needle	0.1	[87]
Hongkong oyster (*C. hongkongensis*) (A)	Soft tissue; 10% KOH (v/v)	5	FT-IR	0.8 ± 0.2	[75]
Densely lamellated oyster (*O. denselamellosa*) (A)	Soft tissue; 10% KOH/30% H_2_O_2_ (v/v)	0.7	Microscope/FT-IR	0.31 ± 0.10	[74]
Japanese scallop (*P. yessoensis*) (M)	Soft tissue; 10% KOH (v/v)	20	Microscope/FT-IR	0.01–0.17	[78]
9 species (M)	Soft tissue; 30% H_2_O_2_ (v/v); NaCl 1.2 g mL^−^^1^	5	Microscope/FT-IR	2.1–10.5	[87]
Manila clam (*T. philippinarum*) (M)	Soft tissue; 10% KOH (v/v)	20	Microscope/FT-IR	0.03–1.08	[78]
Manila clam (*T. philippinarum*) (W)	Soft tissue; 69% HNO_3_ (v/v)	1.2	Microscope	0.9 ± 0.9	[88]
Asian clams (*C. fluminea*) (W)	Soft tissue; 30% H_2_O_2_ (v/v); NaCl 1.2 g mL^−^^1^	20	Microscope/FT-IR	0.3–4.9	[89]
Asian clams (*C. fluminea*) (W)	Soft tissue; 30% H_2_O_2_ (v/v); NaCl 1.2 g mL^−^^1^	5	Microscope/FT-IR/SEM/EDS	0.2–12.5	[90]
*A. squamosus* (W)	Whole sample; 10% KOH (v/v)	38	Microscope/FT-IR	2.89 ± 0.54	[91]
*G.* spp (W)	Whole sample; 10% KOH (v/v)	38	Microscope/FT-IR	0.26 ± 0.08	[91]
Agemaki clam (*S. constricta*) (A)	Soft tissue; 10% KOH/30% H_2_O_2_ (v/v)	0.7	Microscope/FT-IR	0.21 ± 0.05	[74]
Manila clam (*T. philippinarum*) (A)	Soft tissue; 69% HNO_3_ (v/v)	1.2	Microscope	1.7 ± 1.2	[88]
Cockle clam (*C. edule*) (A)	Soft tissue; 10% KOH (v/v)	12	FT-IR	0.74 ± 0.35	[21]
Mud snails (*P. indica*) (W)	Whole body; 10% KOH (v/v)	38	Microscope/FT-IR	3.48 ± 0.89	[91]
common limpet (*P. vulgata*) (W)	Soft tissue; 65% HNO_3_/30% H_2_O_2_	0.7	Microscope/Raman/hot needle test/FT-IR	0–1	[59]
Tower snail (*Turritellidae* sp.) (W)	Soft tissue; 65% HNO_3_/30% H_2_O_2_	0.7	Microscope/Raman/hot needle test/FT-IR	1–4	[59]
Mud snails (*C. cingulate*) (W)	Soft tissue; 30% H_2_O_2_ (v/v)	25	Microscope/FE-SEM/FT-IR/hot needle	1.5	[87]
*Thais mutabilis* (W)	Soft tissue; 30% H_2_O_2_ (v/v)	25	Microscope/FE-SEM/FT-IR/hot needle	2.3	[87]
*Gibbula cineraria* (W)	Soft tissue; 10% KOH (v/v)	0.7	Microscope/FT-IR	3–7/individual	[92]
Common periwinkle (*L. littorea*) (W)	Soft tissue; 65% HNO_3_/30% H_2_O_2_	0.7	Microscope/Raman/hot needle test/FT-IR	1–6	[59]
Common periwinkle (*L. littorea*) (M)	Soft tissue; 65% HNO_3_/30% H_2_O_2_	0.7	Microscope/Raman/hot needle test/FT-IR	27–35	[93]
Common periwinkle (*L. littorea*) (W)	Soft tissue; 10% KOH (v/v)	1.2	Microscope/FT-IR	2.24 ± 3.15	[93]
Brown shrimp (*M. Monoceros*) (W)	Whole body; HNO_3_:HClO_4_ (4:1 v:v)	20	Microscope/hot needle	0.68 ± 0.55	[94]
Australian freshwater shrimp (*P. australiensis*) (W)	Whole body; NaOH 2N	0.45	Microscope/FT-IR	2.4 ± 3.1	[95]
Brown shrimp (*M. Monoceros*) (W)	Soft tissue; 30% H_2_O_2_ (v/v); NaCl 1.2 g mL^−^^1^	45	Microscope/FT-IR	2.17–4.88	[96]
Norway lobster (*N. norvegicus*) (W)	Soft tissue; 69% HNO_3_ (v/v)	-	Microscope/FT-IR	1.75 ± 2.01/individual	[97]
Norway lobster (*N. norvegicus*) (W)	Stomach; 15% H_2_O_2_; NaCl 1.2 g mL^−^^1^	0.45	Microscope/FT-IR	5.5 ± 0.8/individual	[98]
Blue and red shrimp (*A. antennatus*) (W)	Stomach; 15% H_2_O_2_; NaCl 1.2 g mL^−^^1^	0.45	Microscope/FT-IR	1.66 ± 0.11	[98]
Asian tiger shrimp (*P. Monodon*) (W)	Soft tissue; 30% H_2_O_2_ (v/v); NaCl 1.2 g mL^−^^1^	45	Microscope/FT-IR	1.55–4.84	[96]
Spear shrimp (*P. hardwickii*) (A)	Soft tissue; 10% KOH/30% H_2_O_2_ (v/v)	0.7	Microscope/FT-IR	0.25 ± 0.08	[74]
Japanese shore crab (*H. sanguineus*) (W)	Soft tissue; 65% HNO_3_/30% H_2_O_2_	0.7	Microscope/Raman/hot needle test/FT-IR	1–5	[59]
Atlantic blue crab (*C. sapidus*) (W)	Soft tissue; 30% H_2_O_2_ (v/v)	0.8	μFT-IR	0.87/individual	[99]
Atlantic mud crab (*P. herbstii*) (W)	Soft tissue; 30% H_2_O_2_ (v/v)	0.45	Microscope	297.74 ± 1178.75	[85]

n.d.: not detected, ^1^ M: bought from market, W: caught in wild, A: obtained from aquaculture farm.

**Table 4:** Analytical methods and microplastic concentrations in processed foods.

Food Items	Analytical Methods			Concentration (Particles/L or kg)	References
	Digestion/Density Separation	Filtration Pore Size (μm)	Identification		
Beer, USA	Rose Bengal	11	Dissection microscope	n.d.–14.3/L	[32]
Beer, Germany	Rose Bengal	0.8	Dissection microscope	16–254/L	[104]
Honey from 5 countries	30% H_2_O_2_	0.8	Dissection microscope	40–698/kg	[105]
Honey from 9 regions	30% H_2_O_2_	0.8	Microscope	12–418/kg	[106]
Honey, Switzerland	30% H_2_O_2_	30	Microscope	1992–9752/kg (all particles)	[107]
Milks from Mexico, USA, Latin and Central America	Filtration after coagulating lipids	11	Microscope/SEM/Raman	3–11/L	[108]
Sugar	30% H_2_O_2_	0.8	Dissection microscope	249 ± 130/kg	[105]
Teabag, Canada	Distilled water at 95 °C for 5 min	-	SEM/XPS/FT-IR	11.6 billion microplastics (>1 μm) and 3.1 billion nanoplastics (<100 nm in size) per steeped teabag.	[17]
Commercial seaweed nori, China	Cellulase solution (0.1%, v/v), Alcalase solution (100%, v/v), 30% H_2_O_2_ (v/v)/saturated solution of NaCl	5	Stereo optical microscope/FT-IR	0.9–3.0/g (dry weight)	[26]
Canned sardines and sprats from 13 countries	10% KOH/NaI 1.5 g mL^−^^1^	149/8	Microscope/Raman/FESEM-EDX	0–0.75 particles/can	[27]
Dried fish, Malaysia	10% KOH/NaI 1.5 g mL^−^^1^	149/8	Microscope/Raman/FESEM-EDX	0–3 particles/individual fish	[24]
Animal-based traditional medicinal materials, China	30% H_2_O_2_/FeSO_4_·7H_2_O	20	Microscope/FT-IR	1.59 ± 0.33–43.56 ± 9.22/g (dry weight)	[109]

n.d.: not detected.

**Table 5:** Advantages and disadvantages of typical microplastic identification methods.

Identification Method	Advantages	Disadvantages
Visual inspection	Inexpensive, rapid analysis	Possible false-positive detection
Scanning electron microscopy	Not limited to particle size	Possible false-positive detection
Microscopy/FT-IR	Coupled with visual analysis, chemical confirmation of polymers, relatively rapid scanning	Limited to a size of ~20 μm
Microscopy/Raman	Coupled with visual analysis, chemical confirmation of polymers, possible detection to a few micrometers	Time consuming, expensive
Thermal decomposition/GC-MS	Mass measurements, ease of pretreatment	No information about size distribution, potentially biased by large particles, calibration required

**Table 6:** Advantages and disadvantages of typical pretreatment methods used for isolating microplastics.

Pretreatment Method	Applied Matrices	Advantages	Disadvantages	References
*Washing only*	Salts, beer	Very rapid, no need for expensive instruments	Potentials for false-positives, often requires staining	[32,34]
*Oxidative*				
H_2_O_2_	Fish, shellfish, biogenic matter of animal and plant origin	Reduced cost and digestion time, efficient for digesting biological materials	Degradation of PA, PVC, polymethyl methacrylate, and nylon 66; color change of PET	[31,110,111]
Fenton’s reagent	Marine organisms	Good preservation of microplastic particles, effective removal of organic components	To be tested on diverse sample matrices	[112]
*Alkali*				
KOH	Fish, seafood, marine organisms	Effective for destroying proteins, polymer types unaffected with previous environmental degradation	Organic matter such as otoliths, squid beaks, paraffin, and palm fats did not digest; cellulose acetate digested	[113]
NaOH	Seafood, zooplankton, copepods, mussels	Complete digestion of soft tissue, good recovery for PET and HDPE (>97%)	Underrepresentation of pH-sensitive polymers; partial destruction of Nylon, melding of polyethylene, yellowing of uPVC, and loss of several polyester fibers	[19,114]
*Acidic*				
HNO_3_	Seafood, fish, mussels, lugworms	Frozen sample with mild stirring can lead to complete soft tissue digestion in 1 h	Poor results for plastic integrity; decreased particle weight for PA-12, melted LDPE, HDPE, PET, PP; complete destruction of nylon fibers	[19,115,116]
HCl/HNO_3_	Fish	Recovery increased with increasing temperature up to 60 °C	Low digestion efficiency of biological materials (52.5–53.3%)	[31]
HClO_4_	Mussel body and brown shrimp tissues	Stronger perchloric acid reduces the remaining greasy tissue fraction after destruction;lesser effect of HNO_3_ on plastic degradation than other acid digestions	Harmed plastic integrity, sample yellowing	[20,94]
*Enzymatic*				
Corolase 7089 (bacterial protease)	Mussels	Efficient for digesting soft tissue while maintaining microplastic integrity, high recovery (93 ± 10%)	To be tested on different sample types	[19]
Alcalase (industrial protease)	Blue mussel tissue	High digestion efficiency (98.3–99.35%) at low conc.; no visual alterations of PS	Experiments are yet to be conducted using diverse plastic types	[117]
Proteinase-K	Plankton-rich seawater, marine organisms, Antarctic krill	High efficiency, unharmed microplastic debris	Expensive and not suitable for digesting chitin	[110,118]
Trypsin	Mussel tissues	Mild digestion resulting in no change in shape and/or color of polymers	Adductor muscles and mantle skirt were partially digested	[119]
Papain/collagenase	Mussel tissues	No significant changes in exposed polymers	Lower digestive efficacy than trypsin	[119]

## References

[1] Moore C.J. (2008). Synthetic polymers in the marine environment: A rapidly increasing, long-term threat. Environ. Res..

[2] Cózar A., Echevarria F., Gonzákez-Gordillo J.I., Irigoien X., Ubeda B., Hernández-León S., Palma A.T., Navarro S., Garcá-de-Lomas J., Ruiz A. (2014). Plastic debris in the open ocean. Proc. Natl. Acad. Sci. USA.

[3] Eo S., Hong S.H., Song Y.K., Lee J., Lee J., Shim W.J. (2018). Abundance, composition, and distribution of microplastics larger than 20 μm in sand beaches of South Korea. Environ. Pollut..

[4] Ivar do Sul J.A., Costa M.F. (2014). The present and future of microplastic pollution in the marine environment. Environ. Pollut..

[5] Zhang S., Yang X., Gertsen H., Peters P., Salánki T., Geissen V. (2018). A simple method for the extraction and identification of light density microplastics from soil. Sci. Total Environ..

[6] Scheurer M., Bigalke M. (2018). Microplastics in Swiss floodplain soils. Environ. Sci. Technol..

[7] Leslie H.A., Brandsma S.H., van Velzen M.J.M., Vethaak A.D. (2017). Microplastics en route: Field measurements in the Dutch River Delta and Amsterdam Canals, wastewater treatment plants, North Sea sediments and biota. Environ. Int..

[8] Mani T., Hauk A., Walter U., Burkhardt-Holm P. (2015). Microplastics profile along the Rhine River. Sci. Rep..

[9] Park T.-J., Lee S.-H., Lee M.-S., Lee J.-K., Lee S.-H., Zoh K.-D. (2020). Occurrence of microplastics in the Han River and riverine fish in South Korea. Sci. Total Environ..

[10] Park H.-J., Oh M.-J., Kim P.-G., Kim G., Jeong D.-H., Ju B.-K., Lee W.-S., Chung H.-M., Kang H.-J., Kwon J.-H. (2020). National reconnaissance survey of microplastics in municipal wastewater treatment plants in Korea. Environ. Sci. Technol..

[11] Catarino A.I., Macchia V., Sanderson W.G., Thompson R.C., Henry T.B. (2018). Low levels of microplastics (MP) in wild mussels indicate that MP ingestion by humans is minimal compared to exposure via household fibres fallout during a meal. Environ. Pollut..

[12] Akoueson F., Sheldon L.M., Danopoulos E., Morris S., Hotten J., Chapman E., Li J., Rotchell J.M. (2020). A preliminary analysis of microplastics in edible versus non-edible tissues from seafood samples. Environ. Pollut..

[13] Barboza L.G.A., Lopes C., Oliveira P., Bessa F., Otero V., Henriques B., Raimundo J., Caetano M., Vale C., Guilhermino L. (2020). Microplastics in wild fish from North East Atlantic Ocean and its potential for causing neurotoxic effects, lipid oxidative damage, and human health risks associated with ingestion exposure. Sci. Total Environ..

[14] Kim J.-S., Lee H.-J., Kim S.-K., Kim H.-J. (2018). Global pattern of microplastics (MPs) in commercial food-grade salts: Sea salt as an indicator of seawater MP pollution. Environ. Sci. Technol..

[15] Yang D., Shi H., Li L., Li J., Jabeen K., Kolandhasamy P. (2015). Microplastic pollution in table salts from China. Environ. Sci. Technol..

[16] Fadare O.O., Wan B., Guo L.-H., Zhao L. (2020). Microplastics from consumer plastic food containers: Are we consuming it?. Chemosphere.

[17] Hernandez L.M., Xu E.G., Larsson H.C.E., Tahara R., Maisuria V.B., Tufenkji N. (2019). Plastic teabags release billions of microparticles and nanoparticles into tea. Environ. Sci. Technol..

[18] Bouwmeester H., Hollman P.C.H., Peters R.J.B. (2015). Potential health impact of environmentally released micro-and nanoplastics in the human food production chain: Experiences from nanotoxicology. Environ. Sci. Technol..

[19] Catarino A.I., Thompson R., Sanderson W., Henry T.B. (2017). Development and optimization of a standard method for extraction of microplastics in mussels by enzyme digestion of soft tissues. Environ. Toxicol. Chem..

[20] De Witte B., Devriese L., Bekaert K., Hoffman S., Vandermeersch G., Cooreman K., Robbens J. (2014). Quality assessment of the blue mussel (*Mytilus edulis*): Comparison between commercial and wild types. Mar. Pollut. Bull..

[21] Hermabessiere L., Paul-Pont I., Cassone A.-L., Himber C., Receveur J., Jezequel R., El Rakwe M., Rinnert E., Rivière G., Lambert C. (2019). Microplastic contamination and pollutant levels in mussels and cockles collected along the channel coasts. Environ. Pollut..

[22] Abbasi S., Soltani N., Keshavarzi B., Moore F., Turner A., Hassanaghaei M. (2018). Microplastics in different tissues of fish and prawn from the Musa Estuary, Persian Gulf. Chemosphere.

[23] Akhbarizadeh R., Moore F., Keshavarzi B. (2018). Investigating a probable relationship between microplastics and potentially toxic elements in fish muscles from northeast of Persian Gulf. Environ. Pollut..

[24] Karami A., Golieskardi A., Choo C.K., Romano N., Ho Y.B., Salamatinia B. (2017). A high-performance protocol for extraction of microplastics in fish. Sci. Total. Environ..

[25] Neves D., Sobral P., Ferreira J.L., Pereira T. (2015). Ingestion of microplastics by commercial fish off the Portuguese coast. Mar. Pollut. Bull..

[26] Li Q., Feng Z., Zhang T., Ma C., Shi H. (2020). Microplastics in the commercial seaweed nori. J. Hazard. Mater..

[27] Karami A., Golieskardi A., Choo C.K., Larat V., Karbalaei S., Salamatinia B. (2018). Microplastic and mesoplastic contamination in canned sardines and sprats. Sci. Total Environ..

[28] Andrady A.L. (2011). Microplastics in the marine environment. Mar. Pollut. Bull..

[29] Seth C.K., Shriwastav A. (2018). Contamination of Indian sea salts with microplastics and a potential prevention strategy. Environ. Sci. Pollut. Res..

[30] Gündoğdu S. (2018). Contamination of table salts from Turkey with microplastics. Food Addit. Contam. A.

[31] Karami A., Golieskardi A., Choo C.K., Larat V., Galloway T.S., Salamatinia B. (2017). The presence of microplastics in commercial salts from different countries. Sci. Rep..

[32] Kosuth M., Mason S.A., Wattenberg E.V. (2018). Anthropogenic contamination of tap water, beer, and sea salt. PLoS ONE.

[33] Iñiguez M.E., Conesa J.A., Fullana A. (2017). Microplastics in Spanish table salt. Sci. Rep..

[34] Renzi M., Blašković A. (2018). Litter & microplastics features in table salts from marine origin: Italian versus Croatian brands. Mar. Pollut. Bull..

[35] Renzi M., Grazioli E., Bertacchini E., Blašković A. (2019). Microparticles in table salts: Levels and chemical composition of the smallest dimensional fraction. J. Mar. Sci. Eng..

[36] Lee H., Kunz A., Shim W.J., Walther B.A. (2019). Microplastic contamination of table salts from Taiwan, including a global review. Sci. Rep..

[37] Löder M.G.J., Gerdts G., Bergmann M., Gutow L., Klages M. (2015). Methodology used for the detection and identification of microplastics—A critical appraisal. Marine Anthropogenic Litter.

[38] Rochman C.M., Tahir A., Williams S.L., Baxa D.V., Lam R., Miller J.T., Teh F.-C., Werorilangi S., Teh S.J. (2015). Anthropogenic debris in seafood: Plastic debris and fibers from textiles in fish and bivalves sold for human consumption. Sci. Rep..

[39] Cheung L.T.O., Lui C.Y., Fok L. (2018). Microplastic contamination of wild and captive flathead grey mullet (*Mugil cephalus*). Int. J. Environ. Res. Pub. Health.

[40] Jabeen K., Su L., Li J., Yang D., Tong C., Mu J. (2017). Microplastics and mesoplastics in fish from coastal and fresh waters of China. Environ. Pollut..

[41] Avio C.G., Pittura L., d’Errico G., Abel S., Amorello S., Marino G., Gorbi S., Regoli F. (2020). Distribution and characterization of microplastic particles and textile microfibers in Adriatic food webs: General insights for biomonitoring strategies. Environ. Pollut..

[42] Biginagwa F.J., Mayoma B.S., Shashoua Y., Syberg K., Khan F.R. (2016). First evidence of microplastics in the African Great Lakes: Recovery from Lake Victoria Nile perch and Nile tilapia. J. Gt. Lakes Res..

[43] Azizah N., Saragih G.S. (2019). Microplastics in digestive tracts of fishes from Jakarta Bay. IOP Conf. Ser. Earth Environ. Sci..

[44] Zhang D., Cui Y., Zhou H., Jin C., Yu X., Xu Y., Li Y., Zhang C. (2020). Microplastic pollution in water, sediment, and fish from artificial reefs around the Ma’an Archipelago, Shengsi, China. Sci. Total Environ..

[45] Fang C., Zheng R., Chen H., Hong F., Lin L., Lin H., Guo H., Bailey C., Segner H., Mu J. (2019). Comparison of microplastic contamination in fish and bivalves from two major cities in Fujian province, China and the implications for human health. Aquaculture.

[46] Pozo K., Gomez V., Torres M., Vera L., Nuñez D., Oyarzún P., Mendoza G., Clarke B., Fossi M.C., Baini M. (2019). Presence and characterization of microplastics in fish of commercial importance from the Biobío region in central Chile. Mar. Pollut. Bull..

[47] Tanaka K., Takada H. (2016). Microplastic fragments and microbeads in digestive tracts of planktivorous fish from urban coastal waters. Sci. Rep..

[48] Kazour M., Jemaa S., Issa C., Khalaf G., Amara R. (2019). Microplastics pollution along the Lebanese coast (Eastern Mediterranean Basin): Occurrence in surface water, sediments and biota samples. Sci. Total Environ..

[49] Silva-Cacalcanti J.S., Silva J.D.B., de França E.J., de Araújo M.C.B., Gusmão F. (2017). Microplastics ingestion by a common tropical freshwater fishing resource. Environ. Pollut..

[50] Lusher A.L., McHugh M., Thompson R.C. (2013). Occurrence of microplastics in the gastrointestinal tract of pelagic and demersal fish from the English Channel. Mar. Pollut. Bull..

[51] Rummel C.D., Löder M.G.J., Fricke N.F., Lang T., Griebeler E.-M., Janke M., Gerdts G. (2016). Plastic ingestion by pelagic and demersal fish from the North Sea and Baltic Sea. Mar. Pollut. Bull..

[52] Avio C.G., Cardelli L.R., Gorbi S., Pellegrini D., Regoli F. (2017). Microplastics pollution after the removal of the Costa Concordia wreck: First evidences from a biomonitoring case study. Environ. Pollut..

[53] Bucol L.A., Romano E.F., Cabcaban S.M., Siplon L.M.D., Madrid G.C., Bucol A.A., Polidoro B. (2020). Microplastics in marine sediments and rabbitfish (*Siganus fuscescens*) from selected coastal areas of Negros Oriental, Philippines. Mar. Pollut. Bull..

[54] Chagnon C., Thiel M., Antunes J., Ferreira J.L., Sobral P., Ory N.C. (2018). Plastic ingestion and trophic transfer between Easter Island flying fish (*Cheilopogon rapanouiensis*) and yellowfin tuna (*Thunnus albacares*) from Rapa Nui (Easter Island). Environ. Pollut..

[55] De Vries A.N., Govoni D., Árnason S.H., Carlsson P. (2020). Microplastic ingestion by fish: Body size, condition factor and gut fullness are not related to the amount of plastics consumed. Mar. Pollut. Bull..

[56] Foekema E.M., De Gruijter C., Mergia M.T., van Franeker J.A., Murk A.T.J., Koelmans A.A. (2013). Plastic in North Sea fish. Environ. Sci. Technol..

[57] Güven O., Gökdağ K., Jovanović B., Kideyş A.E. (2017). Microplastic litter composition of the Turkish territorial waters of the Mediterranean Sea, and its occurrence in the gastrointestinal tract of fish. Environ. Pollut..

[58] Hermsen E., Pompe R., Besseling E., Koelmans A.A. (2017). Detection of low numbers of microplastics in North Sea fish using strict quality assurance criteria. Mar. Pollut. Bull..

[59] Karlsson T.M., Vethaak A.D., Almroth B.C., Ariese F., van Velzen M., Hassellöv M., Leslie H.A. (2017). Screening for microplastics in sediment, water, marine invertebrates and fish: Method development and microplastic accumulation. Mar. Pollut. Bull..

[60] Liboiron M., Liboiron F., Wells E., Richárd N., Zahara A., Mather C., Bradshaw H., Murichi J. (2016). Low plastic ingestion rate in Atlantic cod (*Gadus morhua*) from Newfoundland destined for human consumption collected through citizen science methods. Mar. Pollut. Bull..

[61] Markic A., Niemand C., Bridson J.H., Mazouni-Gaertner N., Gaertner J.C., Eriksen M., Bowen M. (2018). Double trouble in the South Pacific subtropical gyre: Increased plastic ingestion by fish in the oceanic accumulation zone. Mar. Pollut. Bull..

[62] Mancia A., Chenet T., Bono G., Geraci M.L., Vaccaro C., Munari C., Mistri M., Cavazzini A., Pasti L. (2020). Adverse effects of plastic ingestion on the Mediterranean small-spotted catshark (*Scyliorhinus canicula*). Mar. Environ. Res..

[63] Ogonowski M., Wenman V., Danielsson S., Gorokhova E. Ingested microplastic is not correlated to HOC concentrations in Baltic Sea herring. Proceedings of the 15th International Conference on Environmental Science and Technology.

[64] Koongolla J.B., Lin L., Pan Y.-F., Yang C.-P., Sun D.-R., Liu S., Xu X.-R., Maharana D., Huang J.-S., Li H.-X. (2020). Occurrence of microplastics in gastrointestinal tracts and gills of fish from Beibu Gulg, South China Sea. Environ. Pollut..

[65] Priscilla V., Patria M.P. (2020). Comparison of microplastic abundance in aquaculture ponds of milkfish *Chanos* (Forsskål, 1775) at Muara Kamal and Marunda, Jakarta Bay. IOP Conf. Ser. Earth Environ. Sci..

[66] Boerger C.M., Lattin G.L., Moore S.L., Moore C.J. (2010). Plastic ingestion by planktivorous fishes in the North Pacific Central Gyre. Mar. Pollut. Bull..

[67] Choy C.A., Drazen J.C. (2013). Plastic for dinner? Observations of frequent debris ingestion by pelagic predatory fishes from the central North Pacific. Mar. Ecol. Prog. Ser..

[68] Bellas J., Martínez-Armental J., Martínez-Cámara A., Besada V., Martínez-Gómez C. (2016). Ingestion of microplastics by demersal fish from the Spanish Atlantic and Mediterranean coasts. Mar. Pollut. Bull..

[69] Su L., Deng H., Li B., Chen Q., Pettigrove V., Chenxi W., Shi H. (2019). The occurrence of microplastic in specific organs in commercially caught fishes from coast and estuary area of east China. J. Hazard. Mater..

[70] Huang J.-S., Koongolla J.B., Li H.-X., Lin L., Pan Y.-F., Liu S., He W.-H., Maharana D., Xu X.-R. (2020). Microplastic accumulation in fish from Zhanjiang mangrove wetland, South China. Sci. Total Environ..

[71] Feng Z., Zhang T., Li Y., He X., Wang R., Xu J., Gao G. (2019). The accumulation of microplastics in fish from an important fish farm and mariculture area, Haizhou Bay, China. Sci. Total Environ..

[72] Hurt R., O’Reilly C.M., Perry W.L. (2020). Microplastic prevalence in two fish species in two U.S. reservoirs. Limnol. Oceanogr. Lett..

[73] Fareza A.A., Sembiring E. (2020). Occurrence of microplastics in water, sediment and milkfish (*Chanos chanos*) in Citarum River downstream (Case study: Muara Gembong). E3S Web Conf..

[74] Wu F., Wang Y., Leung J.Y.S., Huang W., Zeng J., Tang Y., Chen J., Shi A., Yu X., Xu X. (2020). Accumulation of microplastics in typical commercial aquatic species: A case study at a productive aquaculture site in China. Sci. Total Environ..

[75] Zhu J., Zhang Q., Li Y., Tan S., Kang Z., Yu X., Lan W., Cai L., Wang J., Shi H. (2019). Microplastic pollution in the Maowei Sea, a typical mariculture bay of China. Sci. Total Environ..

[76] Li Q., Sun C., Wang Y., Cai H., Li L., Li J., Shi H. (2019). Fusion of microplastics into the mussel byssus. Environ. Pollut..

[77] Li J., Green C., Reynolds A., Shi H., Rotchell J.M. (2018). Microplastics in mussels sampled from coastal waters and supermarkets in the United Kingdom. Environ. Pollut..

[78] Cho Y., Shim W.J., Jang M., Han G.M., Hong S.H. (2019). Abundance and characteristics of microplastics in market bivalves from South Korea. Environ. Pollut..

[79] Li J., Yang D., Li L., Jabeen K., Shi H. (2015). Microplastics in commercial bivalves from China. Environ. Pollut..

[80] Li J., Qu X., Su L., Zhang W., Yang D., Kolandhasamy P., Li D., Shi H. (2016). Microplastics in mussels along the coastal waters of China. Environ. Pollut..

[81] Phuong N.N., Poirier L., Pham Q.T., Lagarde F., Zalouk-Vergnoux A. (2018). Factors influencing the microplastic contamination of bivalves from the French Atlantic coast: Location, season and/or mode of life?. Mar. Pollut. Bull..

[82] Mathalson A., Hill P. (2014). Microplastic fibers in the intertidal ecosystem surrounding Halifax Harbor, Nova Scotia. Mar. Pollut. Bull..

[83] Van Cauwenberghe L., Janssen C.R. (2014). Microplastics in bivalves cultured for human consumption. Environ. Pollut..

[84] Martinelli J.C., Phan S., Luscombe C.K., Padilla-Gamiño J.L. (2020). Low incidence of microplastic contaminants in Pacific oysters (*Crassostrea gigas* Thunberg) from the Salish Sea, USA. Sci. Total Environ..

[85] Waite H.R., Donnelly M.J., Walters L.J. (2018). Quantity and types of microplastics in the organic tissues of the eastern oyster *Crassostrea virginica* and Atlantic mud crab *Panopeus herbstii* from a Florida estuary. Mar. Pollut. Bull..

[86] Jahan S., Strezov V., Weldekidan H., Kumar R., Kan T., Sarkodie S.A., He J., Dastjerdi B., Wilson S.P. (2019). Interrelationship of microplastic pollution in sediments and oysters in a seaport environment of the eastern coast of Australia. Sci. Total Environ..

[87] Naji A., Nuri M., Vethaak A.D. (2018). Microplastics contamination in molluscs from the northern part of the Persian Gulf. Environ. Pollut..

[88] Davidson K., Dudas S.E. (2016). Microplastic ingestion by wild and cultured Manila clams (*Venerupis philippinarum*) from Baynes Sound, British Columbia. Arch. Environ. Contam. Toxicol..

[89] Su L., Cai H., Kolandhasamy P., Wu C., Rochman C.M., Shi H. (2018). Using the Asian clam as an indicator of microplastic pollution in freshwater ecosystems. Environ. Pollut..

[90] Su L., Xue Y., Li L., Yang D., Kolandhasamy P., Li D., Shi H. (2016). Microplastics in Taihu Lake, China. Environ. Pollut..

[91] Xu X., Wong C.Y., Tam N.F.Y., Lo H.-S., Cheung S.-G. (2020). Microplastics in invertebrates on soft shores in Hong Kong: Influence of habitat, taxa and feeding mode. Sci. Total Environ..

[92] Jones K.L., Hartl M.G.J., Bell M.C., Capper A. (2020). Microplastic accumulation in a *Zostera marina* L. bed at Deerness Sound, Orkney, Scotland. Mar. Pollut. Bull..

[93] Doyle D., Gammell M., Frias J., Griffin G., Nash R. (2019). Low levels of microplastics recorded from the common periwinkle, *Littorina littorea* on the west coast of Ireland. Mar. Pollut. Bull..

[94] Devriese L.I., van der Meulen M.D., Maes T., Bekaert K., Paul-Pont I., Frère L., Vethaak A.D. (2015). Microplastic contamination in brown shrimp (*Crangon*, Linnaeus 1758) from coastal waters of the Southern North Sea and Channel area. Mar. Pollut. Bull..

[95] Nan B., Su L., Kellar C., Craig N.J., Keough M.J., Pettigrove V. (2020). Identification of microplastics in surface water and Australian freshwater shrimp *Paratya australiensis* in Victoria, Australia. Environ. Pollut..

[96] Hossain M.S., Rahman M.S., Uddin M.N., Sharifuzzaman S.M., Chowdhury S.R., Sarker S., Chowdhury M.S.N. (2020). Microplastic contamination in Penaeid shrimp from the Northern Bay of Bengal. Chemosphere.

[97] Hara J., Frias J., Nash R. (2020). Quantification of microplastic ingestion by the decapod crustacean *Nephrops norvegicus* from Irish waters. Mar. Pollut. Bull..

[98] Cau A., Avio C.G., Dessì C., Follesa M.C., Moccia D., Regoli F., Pusceddu A. (2019). Microplastics in the crustaceans *Nephrops norvegicus* and *Aristeus antennatus*: Flagship species for deep-sea environments?. Environ. Pollut..

[99] Waddell E.N., Lascelles N., Conkle J.L. (2020). Microplastic contamination in Corpus Christi Bay blue crabs, *Callinectes sapidus*. Limn. Oceanogr. Lett..

[100] Munno K., Helm P.A., Jackson D.A., Rochman C., Sims A. (2018). Impacts of temperature and selected chemical digestion methods on microplastic particles. Environ. Toxicol. Chem..

[101] Farrington J.W., Goldberg E.D., Risebrough R.W., Martin J.H., Bowen V.T. (1983). U.S. “Mussel Watch” 1976–1978: An overview of the trace-metal, DDE, PCB, hydrocarbon and artificial radionuclide data. Environ. Sci. Technol..

[102] Monirith I., Ueno D., Takahashi S., Nakada H., Sudaryanto A., Subramanian A., Karuppiah S., Ismail A., Muchtar M., Zheng J. (2003). Asia-Pacific mussel watch: Monitoring contamination of persistent organochlorine compounds in coastal waters of Asian countries. Mar. Pollut. Bull..

[103] Birnstiel S., Soares-Gomes A., da Gama B.A.P. (2019). Depuration reduces microplastic content in wild and farmed mussels. Mar. Pollut. Bull..

[104] Liebezeit G., Liebezeit E. (2014). Synthetic particles as contaminants in German beers. Food Addit. Contam. A.

[105] Liebezeit G., Liebezeit E. (2013). Non-pollen particulates in honey and sugar. Food Addit. Contam. A.

[106] Liebezeit G., Liebezeit E. (2015). Origin of synthetic particles in honeys. Pol. J. Food Nutr. Sci..

[107] Mühlschlegel P., Hauk A., Walter U., Sieber R. (2017). Lack of evidence for microplastic contamination in honey. Food Addit. Contam. A.

[108] Kutralam-Muniasamy G., Pérez-Guevara F., Elizalde-Martínez I., Shruti V.C. (2020). Branded milks—Are they immune from microplastics contamination?. Sci. Total Environ..

[109] Lu S., Qiu R., Hu J., Li X., Chen Y., Zhang X., Cao C., Shi H., Xie B., Wu W.-M. (2020). Prevalence of microplastics in animal-based traditional medicinal materials: Widespread pollution in terrestrial environments. Sci. Total Environ..

[110] Fernández-Severini M.D., Villagran D.M., Buzzi N.S., Sartor G.C. (2019). Microplastics in oysters (*Crassostrea gigas*) and water at the Bahía Blanca Estuary (Southwestern Atlantic): An emerging issue of global concern. Reg. Stud. Mar. Sci..

[111] Sujathan S., Kniggendorf A.K., Kumar A., Roth B., Rosenwinkel K.H., Nogueira R. (2017). Heat and bleach: A cost-efficient method for extracting microplastics from return activated sludge. Arch. Environ. Contam. Toxicol..

[112] Tagg A.S., Harrison J.P., Ju-Nam Y., Sapp M., Bradley E.L., Sinclair C.J., Ojeda J.J. (2017). Fenton’s reagent for the rapid and efficient isolation of microplastics from wastewater. Chem. Comm..

[113] Kühn S., van Franeker J.A., Donoghue A.M.O., Swiers A., Starkenburg M., van Werven B., Lindeboom H. (2020). Details of plastic ingestion and fibre contamination in North Sea. Environ. Pollut..

[114] Cole M., Webb H., Lindeque P.K., Fileman E.S., Halsband C., Galloway T.S. (2014). Isolation of microplastics in biota-rich seawater samples and marine organisms. Sci. Rep..

[115] Dehaut A., Cassone A.L., Frère L., Hermabessiere L., Himber C., Rinnert E., Paul-Pont I. (2016). Microplastics in seafood: Benchmark protocol for their extraction and characterization. Environ. Pollut..

[116] Van Cauwenberghe L., Claessens M., Vandegehuchte M.B., Janssen C.R. (2015). Microplastics are taken up by mussels (*Mytilus edulis*) and lugworms (*Arenicola marina*) living in natural habitats. Environ. Pollut..

[117] Rist S., Steensgaard I.M., Guven O., Nielsen T.G., Jensen L.H., Møller L.F., Hartmann N.B. (2019). The fate of microplastics during uptake and depuration phases in a blue mussel exposure system. Environ. Toxicol. Chem..

[118] Dawson A.L., Kawaguchi S., King C.K., Townsend K.A., King R., Huston W.M., Bengtson Nash S.M. (2018). Turning microplastics into nanoplastics through digestive fragmentation by Antarctic krill. Nat. Commun..

[119] Courtene-Jones W., Quinn B., Murphy F., Gary S.F., Narayanaswamy B.E. (2017). Optimisation of enzymatic digestion and validation of specimen preservation methods for the analysis of ingested microplastics. Anal. Methods.

[120] Mintenig S.M., Int-Veen I., Löder M.G.J., Primpke S., Gerdts G. (2017). Identification of microplastic in effluents of waste water treatment plants using focal plane array-based micro-Fourier-transform infrared imaging. Water Res..

[121] Löder M.G.J., Imhof H.K., Ladehoff M., Löschel L.A., Lorenz C., Mintenig S., Piehl S., Primpke S., Schrank I., Laforsch C. (2017). Enzymatic porification of microplastics in environmental samples. Environ. Sci. Technol..

[122] Nuelle M.-T., Dekiff J.H., Remy D., Fries E. (2014). A new analytical approach for monitoring microplastics in marine sediments. Environ. Pollut..

[123] Fries E., Dekiff J.H., Willmeyer J., Nuelle M.-T., Ebert M., Remy D. (2013). Identification of polymer types and additives in marine microplastic particles using pyrolysis-GC/MS and scanning electron microscopy. Environ. Sci. Process. Impacts.

[124] Fischer M., Scholz-Böttcher B.M. (2017). Simultaneous trace identification and quantification of common types of microplastics in environmental samples by pyrolysis-gas chromatography-mass spectrometry. Environ. Sci. Technol..

[125] Hermabessiere L., Himber C., Boricaud B., Kazour M., Amara R., Cassone A.-L., Laurentie M., Paul-Pont I., Soudant P., Dehaut A. (2018). Optimization, performance, and application of a pyrolysis-GC/MS method for the identification of microplastics. Anal. Bioanal. Chem..

[126] Kang H.-J., Park H.-J., Kwon O.-K., Lee W.-S., Jeong D.-H., Ju B.-K., Kwon J.-H. (2018). Occurrence of microplastics in municipal sewage treatment plants: A review. Environ. Health Toxicol..

[127] Qiao R., Deng Y., Zhang S., Wolosker M.B., Zhu Q., Ren H., Zhang Y. (2019). Accumulation of different shapes of microplastics initiates intestinal injury and gut microbiota dysbiosis in the gut of zebrafish. Chemosphere.

[128] Ziajahromi S., Kumar A., Neale P.A., Leusch F.D.L. (2017). Impact of microplastic beads and fibers on waterflea (*Ceriodaphnia dubia*) survival, growth, and reproduction: Implications of single and mixture exposures. Environ. Sci. Technol..

[129] Au S.Y., Bruce T.F., Bridges W.C., Klaine S.J. (2015). Responses of *Hyalella azteca* to acute and chronic microplastic exposures. Environ. Toxicol. Chem..

[130] Thiele C.J., Hudson M.D., Russell A.E. (2019). Evaluation of existing methods to extract microplastics from bivalve tissue: Adapted KOH digestion protocol improves filtration at single-digit pore size. Mar. Pollut. Bull..

[131] Käppler A., Fischer D., Oberbeckmann S., Schernewski G., Labrenz M., Eichhorn K.-J., Voit B. (2016). Analysis of environmental microplastics by vibrational microspectroscopy: FTIR, Raman or both?. Anal. Bioanl. Chem..

[132] Renner G., Schmidt T.C., Schram J. (2020). Automated rapid & intelligent microplastics mapping by FTIR microscopy: A Python-based workflow. MethodsX.

[133] Primpke S., Lorenz C., Rascher-Friesenhausen R., Gerdts G. (2017). An automated approach for microplastics analysis using focal plane array (FPA) FTIR microscopy and image analysis. Anal. Methods.

[134] Kögel T., Bjorøy Ø., Toto B., Bienfait A.M., Sanden M. (2020). Micro-and nanoplastic toxicity on aquatic life: Determining factors. Sci. Total Environ..

[135] Yong C.Q.Y., Valiyaveetill S., Tang B.L. (2020). Toxicity of microplastics and nanoplastics in mammalian systems. Int. J. Environ. Res. Public Health.

[136] Peeken I., Primpke S., Beyer B., Gütermann J., Katlein C., Krumpen T., Bergmann M., Hehemann L., Gerdts G. (2018). Arctic Sea ice is an important temporal sink and means of transport for microplastic. Nat. Commun..

[137] Enders K., Lenz R., Stedmon C.A., Nielsen T.G. (2015). Abundance, size and polymer composition of marine microplastics ≥10 μm in the Atlantic Ocean and their modelled vertical distribution. Mar. Pollut. Bull..

[138] Pedrotti M.L., Petit S., Elineau A., Bruzaud S., Crebassa J.C., Dumontet B., Martí E., Gorsky G., Cózar A. (2016). Changes in the floating plastic pollution of the Mediterranean Sea in relation to the distance to land. PLoS ONE.

[139] Magri D., Sánchez-Moreno P., Caputo G., Gatto F., Veronesi M., Bardi G., Catelani T., Guarnieri D., Athanassiou A., Pompa P.P. (2018). Laser ablation as a versatile tool to mimic polyethylene terephthalate nanoplastic pollutants: Characterization and toxicology assessment. ACS Nano.

[140] Gasperi J., Wright S.L., Dris R., Collard F., Mandin C., Guerrouache M., Langlois V., Kelly F.J., Tassin B. (2018). Microplastics in air: Are we breathing it in?. Curr. Opinion Environ. Sci. Health.

[141] Vianello A., Jensen R.L., Liu L., Vollertsen J. (2019). Simulating human exposure to indoor airborne microplastics using a Breathing Thermal Manikin. Sci. Rep..

